# Satellite Observations of Imprint of Oceanic Current on Wind Stress by Air-Sea Coupling

**DOI:** 10.1038/s41598-017-17939-1

**Published:** 2017-12-18

**Authors:** Lionel Renault, James C. McWilliams, Sebastien Masson

**Affiliations:** 1 Department of Atmospheric and Oceanic Sciences, University of California, Los Angeles, California USA; 2 LEGOS, Université de Toulouse, IRD, CNRS, CNES, UPS,, Toulouse, France; 3 Sorbonne Universites (UPMC, Univ Paris 06)-CNRS-IRD-MNHN, LOCEAN Laboratory, 4 place Jussieu, 75005 Paris, France

## Abstract

Mesoscale eddies are present everywhere in the ocean and partly determine the mean state of the circulation and ecosystem. The current feedback on the surface wind stress modulates the air-sea transfer of momentum by providing a sink of mesoscale eddy energy as an atmospheric source. Using nine years of satellite measurements of surface stress and geostrophic currents over the global ocean, we confirm that the current-induced surface stress curl is linearly related to the current vorticity. The resulting coupling coefficient between current and surface stress (s_τ_ [N s m^−3^]) is heterogeneous and can be roughly expressed as a linear function of the mean surface wind. s_τ_ expresses the sink of eddy energy induced by the current feedback. This has important implications for air-sea interaction and implies that oceanic mean and mesoscale circulations and their effects on surface-layer ventilation and carbon uptake are better represented in oceanic models that include this feedback.

## Introduction

**Figure 1:**
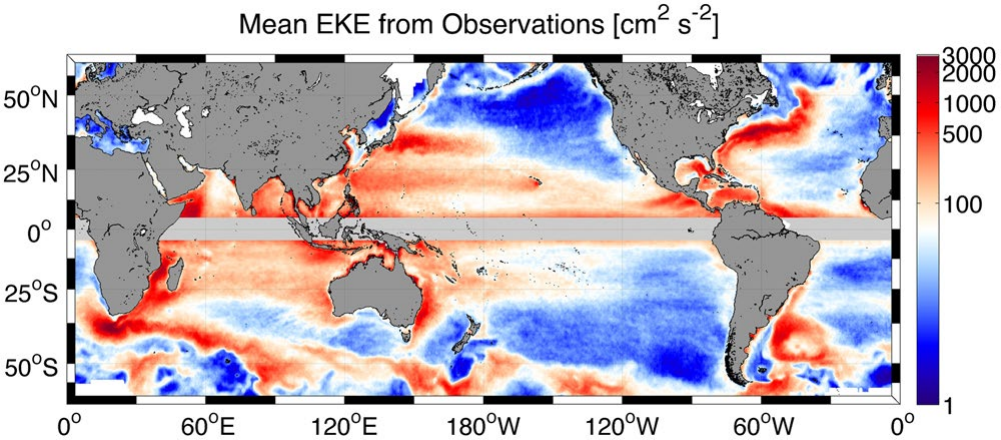
Global satellite observations allow monitoring mesoscale oceanic currents as illustrated here by the *EKE* estimated from the AVISO geostrophic currents. The gray color masks the equatorial region where geostrophic approximation is not reliable. The Figure has been generated using Matlab R2014b (https://www.mathworks.com/) and E.U. Copernicus Marine Service Information data (AVISO).

Mesoscale eddies, generated by baroclinic and barotropic instabilities of the persistent currents, are present everywhere in the world ocean and play a key role in many oceanic processes. Their description and understanding have been improved in the last decades due to the development and the use of satellite missions and high-resolution numerical simulations. The Eddy Kinetic Energy (estimated as $EKE=\overline{0.5({u}^{\text{'}2}+{v}^{\text{'}2})}$ [m^2^ s^−2^]) as a measure of the intensity of the mesoscale activity is computed here from the geostrophic currents anomalies derived from the high-pass filtered (running mean over 91 days) AVISO product (EU Copernicus Marine Service) over the period 2000–2008. Consistent with previous studies (*e.g*.,^1^), Western Boundary Currents (WBC) and the Antarctic Circumpolar Current (ACC) are the most eddy active regions (Fig. 1). Eastern Boundary Currents, although less active, have relatively high *EKE* compared to the offshore ocean. In the WBC, the mesoscale activity is known to have a large impact on the mean currents as the Gulf Stream (*e.g*.,^2,3^), the Agulhas Current Retroflection (*e.g*.,^4–7^), and the Kuroshio (*e.g*.,^8,9^) and in general on the primary production^10^. The Eastern Boundary Currents are known to be very sensitive to the mesoscale activity as it can strongly modulate the primary production^11,12^, and the offshore transport of heat and biogeochemical materials^13,14^. In the Southern Ocean, the eddy activity has substantial implication for total transport and the uptake of carbon and heat^15,16^. Understanding and representing the mesoscale activity in numerical model is therefore of great importance.

The ocean can couple with the atmosphere both through the oceanic thermal feedback (*e.g*.,^17–24^) and the current feedback (*e.g*.,^25–34^). Both coupling processes strongly involve mesoscale eddies. At the mesoscale, Sea Surface Temperature (SST) induces a clear imprint on the surface stress, *e.g*.,^17^ empirically show the presence of linear relationships between the crosswind (downwind) components of the local sea surface temperature gradient and the surface stress curl (divergence)^17^. Forerunner studies such as^25^ and^26^ analytically show the current feedback should systematically surface stress anomalies that can be approximated by a linear function of oceanic surface current. The surface stress as determined by the QuikSCAT satellite already incorporates these feedbacks^17,35^. Under limited circumstance such as an eddy-centric framework^34^, show the SST mesoscale effect on the surface stress is usually secondary to the current feedback. Observational and numerical studies have highlighted some effects of mean oceanic currents on the mean surface stress^35^, using the Tropical Atmosphere-Ocean (TAO) and satellite scatterometer data, show the current feedback reduces the median wind stress magnitude by 20%. By reducing the energy input from the atmosphere to the ocean, the current feedback slows down the mean oceanic currents^36,37^, and partially controls the WBC^7,32^. It also induces a dampening of the mesoscale activity via an “eddy killing”, *i.e*., a sink of energy from eddies to the atmosphere^31^.

In this study, using nine years of satellite measurements of surface stress and geostrophic currents over the global ocean, the focus is on the characterization of the effect of the surface currents on the surface stress and on the exchange of energy between the oceanic mesoscale and the atmosphere. Specifically, the objectives are (i) to determine at a global scale the spatial and temporal variability of the coupling coefficient between surface current vorticity and stress curl, (ii) to assess the main parameters that drive such a variability, and (iii) to determine its consequence on the exchange of energy between the oceanic mesoscale and the atmosphere. The implication on how to force an ocean model and a tentative parameterization of the current feedback for a forced ocean model are also discussed.

## Results

The surface stress can be represented in a bulk formulae by using the difference of the wind relative to the current:

1 $$\tau ={\rho }_{a}{C}_{D}({{U}}_{a}-{{U}}_{o})|{{U}}_{a}-{{U}}_{o}|\,,$$

where τ is the surface stress, *ρ*_*a*_ is the density of the air, *C*_*D*_ is the drag coefficient, and *U*_*a*_ and *U*_*o*_ are the 10 m wind and the surface current, respectively. When neglecting the current feedback, under the same assumptions the stress is estimated as

2 $${\tau }_{a}={\rho }_{a}{C}_{D}{{U}}_{a}|{{U}}_{a}|\,\mathrm{.}$$

Following^25,26,28,34^, at mesoscale, if we assume that |*U*_*o*_| $\ll$ |*U*_*a*_|, the stress difference between (1) and (2) ($\tau {^{\prime} }_{diff}$) can be approximated as (The details of the derivation are given in SI):

3 $$\tau {^{\prime} }_{diff}\sim -\frac{3}{2}{\rho }_{a}{C}_{D}\,|{{U}}_{a}|\,{{U}}_{o}^{\prime} \mathrm{.}$$

where ${{U}}_{o}^{\prime}$ represents the mesoscale oceanic currents. Recently, focusing on the U.S. West coast with ocean-atmosphere coupled simulations^31^, show at mesoscale the surface stress response to the current feedback can also be expressed through a regression coefficient *s*_*τ*_ as

4 $$\tau {^{\prime} }_{diff}={s}_{\tau }{{U}}_{o}^{\prime} ,$$

Equating these two expressions gives a relation where *s*_*τ*_ is a linear function of the wind:

5 $${s}_{\tau }\sim -\frac{3}{2}{\rho }_{a}{C}_{D}|{{U}}_{a}|$$

Assuming nominal values of the constants in (5) with a *C*_*D*_ = 1.2 10^−3^ and a *ρ*_*a*_ = 1.225 kg m^−3^:

6 $${s}_{\tau }\sim -2.20\,{10}^{-3}\,N\,{m}^{-4}\,{s}^{2}\,|{{U}}_{a}\mathrm{|.}$$

**Figure 2:**
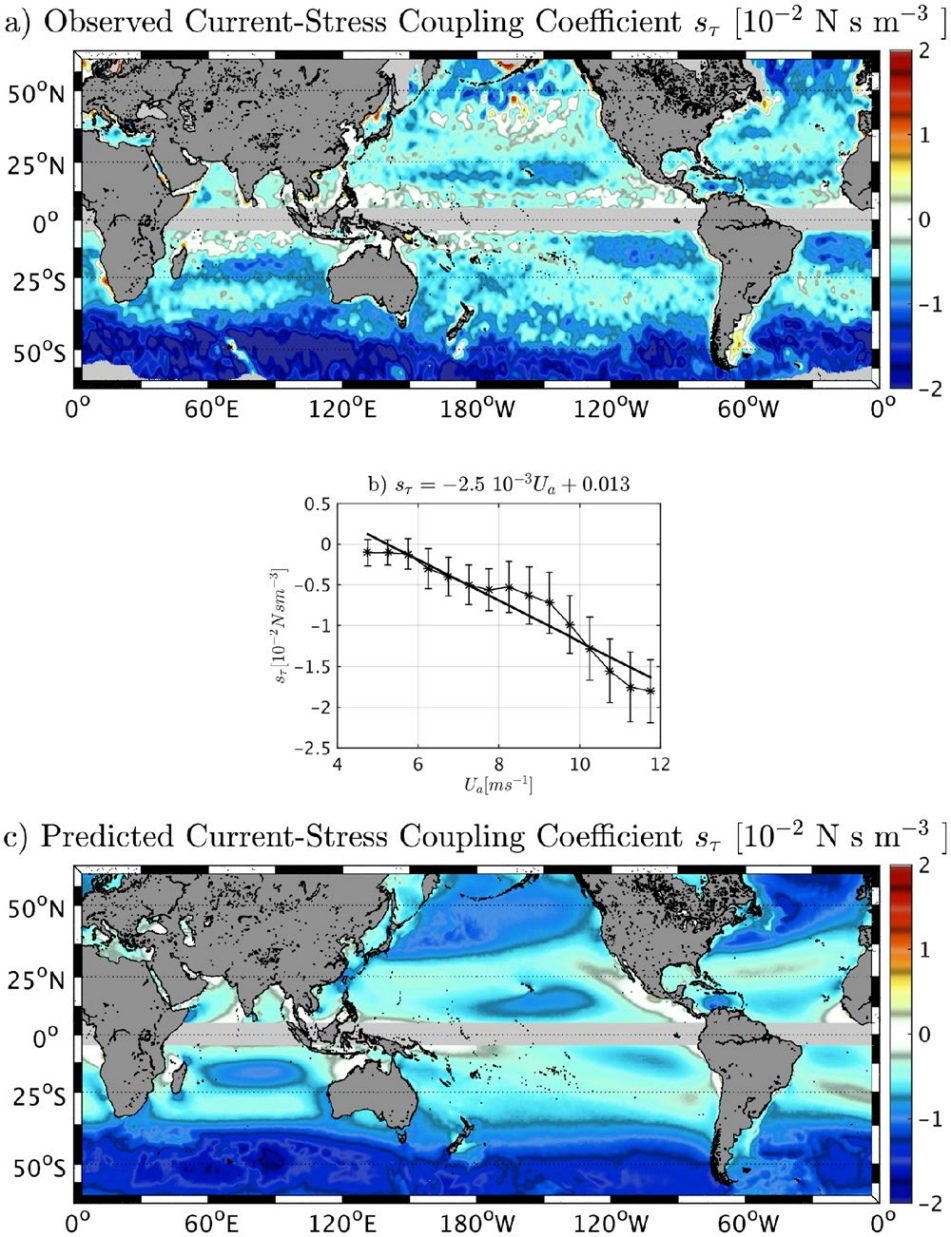
The current feedback to the atmosphere induces persistent surface stress anomalies that can be expressed as a linear relationship. It causes a sink of energy from geostrophic currents. (**a**) Coupling coefficient *s*_*τ*_ between surface geostrophic current and surface stress. (**b**) Binned scatterplot of the full time series of 10m-wind magnitude and *s*_*τ*_ over the World Ocean. The bars indicate plus and minus one standard deviation about the mean marked by stars. The linear regression is indicated by a black line, and the slope is indicated in the title. (**c**) Predicted *s*_*τ*_ = (−2.5 10^−3^|*U*_*a*_| + 0.013 m s^−1^) N s ^2^ m ^−4^ (see text). The Figure has been realized using Matlab R2014b (https://www.mathworks.com/) and data from QuikSCAT V3 product (CERSAT, IFREMER) and E.U. Copernicus Marine Service Information data (AVISO).

The use of derivatives of surface stress and currents allows to efficiently isolate the current feedback effect on the surface stress from the SST feedback^31^. Therefore, to quantify the effect of the mesoscale surface current on the surface stress at a global scale, the coupling coefficient *s*_*τ*_ ([N s m^−3^]) is defined as the slope of the linear regression at each grid point between monthly average and spatially filtered (see SI for more details) geostrophic surface vorticity (from AVISO) and surface stress curl (from a QuikSCAT product^38^) over the whole altimeter-scatterometer overlap period (2000–2008) and also by seasons (not shown). (Note that measurements closer than 100 km to the coast may have a substantial effect of the orography and the coastline on the wind^19,39^.) The resulting global map is slightly smoothed (over 50 km) to diminish sampling noise due to the relatively short analysis period (9 years). The mesoscale surface currents systematically induce persistent surface stress anomalies everywhere (Fig. 2a). *s*_*τ*_ is characterized by a large-scale variability. The high-latitude regions have the largest *s*_*τ*_. Eddies in those regions should therefore be strongly damped by the current feedback. Eastern boundary currents have medium range values of *s*_*τ*_ around 1 *N s m* ^−3^, slightly weaker than the value found by^31^ for the U.S. West Coast using numerical coupled model. While no doubt part of the discrepancy may be due to model bias, it could also be explained by uncertainties in the observations as discussed in the last section. There are a few regions where *s*_*τ*_ appears positive, although it could be due to uncertainties in the observations, this could also indicate regions where wind variations force weak eddy variability (Fig. 1).

The linear relationship between wind magnitude and *s*_*τ*_ is confirmed by analyzing the *s*_*τ*_ global values in Fig. 2a and by comparing it to a mean 10m-wind map (not shown). Regions characterized by a mean large wind have an strong imprint of the current on the surface stress, and, thus, have a large *s*_*τ*_. The primary dependence of *s*_*τ*_ on the wind is furthermore corroborated by analyzing the statistical relationship between mean 10m-wind and *s*_*τ*_. Global bin-averaged values of 10m-wind magnitude (bins of 0.1 m s^−1^) and *s*_*τ*_ are computed over the whole period (2000–2008, Fig. 2b). They have a clear negative linear relationship (*σ* > 0.95 using a t-test):

7 $${s}_{\tau }=-2.5\,{10}^{-3}\,N{m}^{-4}{s}^{2}|{U}_{a}|\,+\,0.013\,N\,{m}^{-3}\,s,$$

The expression in (6) has implicit further dependencies in *C*_*D*_ on *U*_*a*_ and on wave age^40–42^, so that imperfections in the observational regression fit of (7) can partly be attributed to these other dependencies, to the *θ* approximation (see SI), and also to a possible partial re-energization of the eddies by the wind response to the current feedback^31^. Interestingly, a similar relationship has been found by^43^ for the SST coupling coefficient that also primarily depends on the mean wind distribution.

A predicted *s*_*τ*_ is then computed in Fig. 2c using (7). The mean 10m-wind magnitude appears to be a fair predictor of *s*_*τ*_, indicating its primary role in determining its spatial variation. *s*_*τ*_ is characterized by a seasonal cycle that is mainly driven by the 10m-wind seasonal cycle (not shown). The surface stress response to the current feedback can have furthermore dependencies. For example, in (1), a second order term (because *U*_*a*_ $\gg$ *U*_*o*_) can be derived as: ${\rho }_{a}{C}_{D}{{U}}_{o}^{2}$. The secondary importance of this term is confirmed by the less obvious relationship that can be found between the logarithm of the *EKE* and *s*_*τ*_: the larger a $\mathrm{log}\,[EKE]$, the larger a *s*_*τ*_ (not shown). However, the statistical relationship between *EKE* and *s*_*τ*_ represents only *s*_*τ*_ values between −0.8 10^−2^ and −1.5 10^−2^ N s m^−3^ and has a large spread. Additionally, as a second order effect of the current feedback, the wind response to the current feedback partially damps the surface stress changes and thus weakens *s*_*τ*_^31^. The wind response depends on the marine boundary layer height *h*: the shallower *h*, the larger a wind response^7^, and, thus, the weaker *s*_*τ*_. However, no significant relationship between the mean *h* from ERA interim^44^ and *s*_*τ*_ has been found.

**Figure 3:**
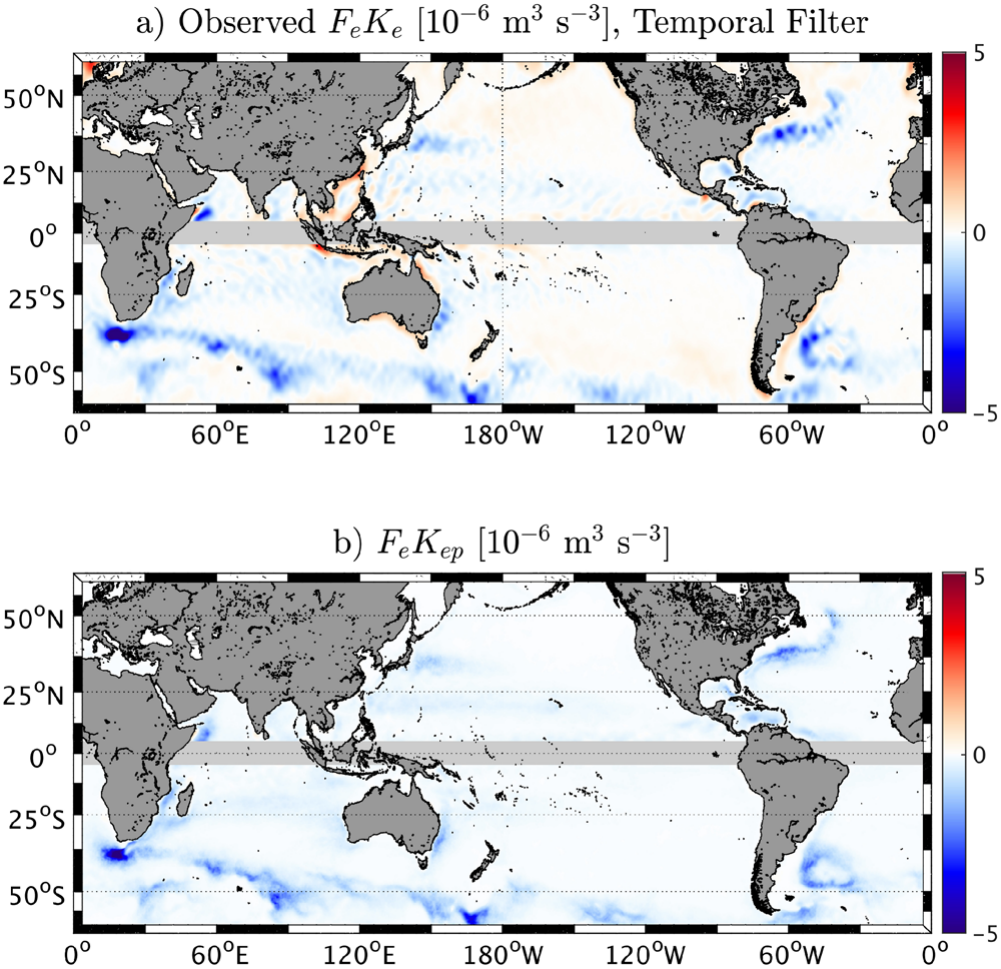
(**a**) Mean eddy wind work (*F*_*e*_ *K*_*e*_) estimated using a temporal filter (91 days). (**b**) Predicted *F*_*e*_ *K*_*ep*_ = (2)/(*ρ*_*o*_)*s*_*τ*_ *EKE* using the seasonal values of *s*_*τ*_ and *EKE*. A negative *F*_*e*_ *K*_*e*_ indicates a transfer of energy from the oceanic eddies to the atmosphere. It induces a damping of the eddies. The Figure has been realized using Matlab R2014b (https://www.mathworks.com/) and data from QuikSCAT V3 product (CERSAT, IFREMER) and E.U. Copernicus Marine Service Information data (AVISO).

As shown by *e.g*.,^29,30,32,45,46^, a direct effect of the current feedback is to transfer energy from mesoscale eddies to the atmosphere. The geostrophic eddy wind work (*F*_*e*_ *K*_*e*_ [m ^3^ s^−3^]) expresses the transfer of kinetic energy between the atmospheric wind and oceanic eddies:

8 $${F}_{e}{K}_{e}=\frac{1}{{\rho }_{0}}\,(\overline{{\tau }_{x}^{^{\prime} }\,{u}_{og}^{^{\prime} }}+\overline{{\tau }_{y}^{^{\prime} }\,{v}_{og}^{^{\prime} }}),$$

where prime denotes the eddy part of the signal usually estimated using a temporal filter^47^ (here using a running 91-day window), *ρ*_0_ is the ocean surface density, *τ*_*x*_ and *τ*_*y*_ are the zonal and meridional surface stresses, and *u*_*og*_ and *v*_*og*_ are the zonal and meridional geostrophic currents. Figure 3a shows the *F*_*e*_ *K*_*e*_ estimated using geostrophic currents from AVISO and surface stress from QuikSCAT over the period 2000–2008. Consistent with previous studies (*e.g*.,^29,47^), this estimate reveals large-scale pathways of energy from the oceanic eddies to the atmosphere that induce a damping of the mesoscale activity by ≈30 %^7,27,28,31–33^. The most mesoscale active regions (*e.g*., WBC and ACC) have the largest negative eddy wind work. The total *F*_*e*_ *K*_*e*_ away of the tropics (excluding 5°S-5°N) is ≈−23 GW, which is consistent with *e.g*., the^47^ estimate (based also on a temporal filter). Along the coast the wind perturbations induce an oceanic coastal jet that flows partially in the same direction as the wind^48^, inducing a positive *F*_*e*_ *K*_*e*_. The offshore weakly positive values of *F*_*e*_ *K*_*e*_ are regions where the *EKE* is very weak and likely the wind forces the local surface currents^47,49^.

In light of the coupling coefficient between the surface current and the surface stress *s*_*τ*_ and eq. (8) and (4), a predicted *F*_*e*_ *K*_*ep*_ is estimated as the product of the seasonal values of *s*_*τ*_ and *EKE* (Fig. 3b):

9 $${F}_{e}{K}_{ep}=\frac{2}{{\rho }_{o}}{s}_{\tau }EKE$$

The sink of energy from the geostrophic current to the atmosphere mainly depends on the *EKE*, however it is modulated by *s*_*τ*_: the more negative a *s*_*τ*_, the more efficient an eddy killing effect and, hence, larger a sink of eddy energy. *s*_*τ*_ can be interpreted as a measure of the efficiency of the current feedback. Regions characterized by a weak *EKE* (*e.g*., center of North Pacific) may have a large *s*_*τ*_, but their sink of energy remain very weak. The WBC and in particular the ACC, characterized by both an important mesoscale activity and a large mean wind, are regions with the largest sink of energy. Eastern boundary currents are characterized by a weaker sink of energy than WBC because of their milder EKE. The total predicted *F*_*e*_ *K*_*ep*_ away of the tropics is ≈−48 GW, which is much larger than an estimate based on Fig. 3a. This estimate can be interpreted as a direct measure of the transfer of energy from the mesoscale geostrophic currents to the atmosphere induced by the current feedback whereas an estimate based on a Reynolds decomposition does not capture only the atmospheric response to the current feedback but all kind of “eddy windwork” (*e.g*., wind-driven currents). *F*_*e*_ *K*_*e*_ can furthermore be estimated by considering the current and stress anomalies using a high-pass Gaussian spatial filter with a 250 km cut-off (see SI). This estimate is closer to the predicted *F*_*e*_ *K*_*ep*_ (not shown) and represents a total sink of energy away of the tropic by ≈70*GW*, confirming the difference between a Reynolds decomposition estimate of *F*_*e*_ *K*_*e*_ and the predicted *F*_*e*_ *K*_*ep*_. *F*_*e*_ *K*_*e*_ estimated using a spatial filter is larger than the other *F*_*e*_ *K*_*e*_ estimates because 1) wind driven currents (that induces positive *F*_*e*_ *K*_*e*_) have a larger scale than the oceanic mesoscale and, thus, are not included when estimating the wind work using a spatial filter; and 2) the *F*_*e*_ *K*_*e*_ estimated using a spatial filter also includes the effect of strong currents (such as the Gulf Stream) on the surface stress that causes an additional sink of energy from the ocean to the atmosphere.

## Discussion

The main effect of the current feedback at the mesoscale is to induce a negative *F*_*e*_ *K*_*e*_ (≈−48 GW), indicating a sink of energy from the mesoscale currents to the atmosphere^47^. suggest 760 GW is an upper limit on the total wind work. The total wind work is much larger than *F*_*e*_ *K*_*e*_ because it also includes the mean wind work. The mean wind work represents the transfer of energy from mean surface wind forcing to mean kinetic energy, it is the main driver of the oceanic circulation and an important energy sink for the atmosphere. However, understanding and representing *F*_*e*_ *K*_*e*_ is crucial at least for ocean modeling and apprehending the energy budget of the ocean, because it represents a large dampening of mesoscale activity. The energy transfer to the atmosphere may cause an adjustment of the wind that in turn partly counteracts the stress effect and partially re-energizes the ocean^31^. However, from an atmospheric point of view the wind changes are rather small, *e.g*., for the US West Coast a current of 1 ms^−1^ induces a wind anomaly of ≈0.2 ms^−1^ ^31^.

The substantial current feedback effect on the currents should change the paradigm of how to force an regional high resolution uncoupled oceanic model. However, regional models and even global reanalysis (NCEP or ERA) generally still ignore this feedback. When forcing an ocean model with an atmospheric product that does not contain the atmospheric response to the current feedback (as *e.g*., NCEP but not as QuikSCAT), this effect could be incorporated by using in the bulk formulae the relative wind to the current (instead of the wind alone) with a parameterization of the wind response that partially re-energizes the ocean^31^. suggest using a simple wind correction to make to a wind *U*_*a*_ to mimic the coupled response in an uncoupled oceanic model. Such a parameterization is based on the current-wind coupling coefficient *s*_*w*_ estimated from a coupled simulation. A different parameterization could be based on a stress correction to make a τ that mimics the coupled surface stress response (*i.e*., that includes the wind adjustment) in an uncoupled oceanic model, *viz*.,

10 $$\tau ={\tau }_{a}+{s}_{\tau }{{U}}_{o}\mathrm{.}$$

When forcing an ocean model, this correction could be applied on a prescribed surface stress or to a surface stress estimated using a bulk formulae and the absolute wind. We intend to investigate this further.

Both datasets used in this study have limitations mainly due to their effective spatial resolution. There are eddies in the ocean scales smaller than can be resolved by the AVISO dataset (*i.e*., with radius bigger than about 40 km^50,51^). The QuikSCAT product used in this study has a spatial resolution of 0.25°, but an effective resolution of about 1° ^52^. As a results, although the coupling coefficient between current and stress (*s*_*τ*_) is mainly driven by the mean surface wind, its empirical estimation depends on the methodology used, the observations, and, thus, suffers from uncertainties. For example, the geostrophic currents may be underestimated because of the smoothness of AVISO. This would tend to overestimate *s*_*τ*_ because the observed stress response would correspond to larger currents (see Fig. S2,SI). Finally, QuikSCAT contains mesoscale structure induced by both currents and SST^20,43^. The coupling coefficients estimated here and by *e.g*.,^20^, could therefore be somehow influenced by other feedbacks, such as the thermal feedback. However, as shown by^31^, estimating *s*_*τ*_ using the the surface stress curl and the surface vorticity efficiently allows to isolate the stress response to the surface currents from the stress induced by the SST feedback. Finally, it is worth noting the large-scale sink of energy from mesoscale currents to the atmosphere are not induced by the SST feedback but only by the current feedback. Indeed a coupled simulation that takes into account only the thermal feedback has a *F*_*e*_ *K*_*e*_ ≈ 0 (except along the coast where is it positive), whereas a coupled simulation that considers both thermal and current feedbacks is characterized offshore by a negative *F*_*e*_ *K*_*e*_^7^. Further studies, based on coupled numerical simulations, should aim to properly isolate the different feedbacks.

The mechanism of mesoscale transfer of energy from the ocean to the atmosphere associated with the damping of eddies is valid for most of the ocean, especially for the Western Boundary Current and the Southern Ocean. This mechanism is likely crucial to understand how eddies affect the mean circulation but also shape the Oxygen Minimum Zones (*e.g*.,^53^) and carbon uptake (*e.g*.,^54^). It is also likely to be relevant to future climate changes involving the oceanic meridional overturning circulation because of its dependency on eddy fluxes (especially in the ACC;^55^).

## Electronic supplementary material

Supplementary Information
