# Supplementary material for: Satellite Observations of Imprint of Oceanic Current on Wind Stress by Air-Sea Coupling
Source: Sci Rep. 2017 Dec 18;7:17747. doi: 10.1038/s41598-017-17939-1 (PMC5735162; doi:10.1038/s41598-017-17939-1)
Supplement: Supplementary file 1 — Supplementary Information [file 41598_2017_17939_MOESM1_ESM.pdf]

# Satellite Observations of Imprint of Oceanic Current on Wind Stress by Air-Sea Coupling - Supplemental Information

November 16, 2017

By Lionel Renault, James C. McWilliams, Sebastien Masson

## 1. Methods

*a. Temporal Filter*

The temporal filter is formed by subtracting a 91-day running mean of a field $\phi$: $\phi' = \phi - \overline{\phi}_{91days}$, with $\overline{\phi}_{91days}$ the 91-day running mean of $\phi$.

*b. Spatial Filter*

When using a spatial filter, a field $\phi$ is smoothed using a Gaussian spatial filter with a standard deviation of 4. Gaussian weight of points located at a distance larger than $3\sigma$ are considered zero. The Gaussian filter is thus applied on a $(6\sigma + 1)$ x $(6\sigma + 1)$ window which makes 25x25 points ($\approx 6°$x 6 °) in our case. The cutoff of this filter is about $250km$. Land points are treated as missing data and the weights of windows including land points are renormalized over the remeaning oceanic points. Mesoscale anomalies of a field $\phi$ are then defined as $\phi' = \phi - [\phi]$, with $[\phi]$ the smoothed field.

*c. Approximation of the current feedback induced surface stress*

In a bulk formulae, the surface stress can be represented by using the difference of the wind relative to the current:

$$\boldsymbol{\tau} = \rho_a C_D (\boldsymbol{U}_a - \boldsymbol{U}_o) |\boldsymbol{U}_a - \boldsymbol{U}_o| \,, \tag{1}$$

where $\boldsymbol{\tau}$ is the surface stress, $\rho_a$ is the density of the air, $C_D$ is the drag coefficient, and $\boldsymbol{U}_a$ and $\boldsymbol{U}_o$ are the 10 m wind and the surface current, respectively. When neglecting the current feedback, under the same assumptions the stress is estimated as

$$\boldsymbol{\tau}_a = \rho_a C_D \boldsymbol{U}_a |\boldsymbol{U}_a| \,. \tag{2}$$

The surface stress anomalies induced by the current feedback can be represented as:

$$\boldsymbol{\tau}_{diff} = \boldsymbol{\tau} - \boldsymbol{\tau}_a = \rho_a C_D ((\boldsymbol{U}_a - \boldsymbol{U}_o)|\boldsymbol{U}_a - \boldsymbol{U}_o| - \boldsymbol{U}_a |\boldsymbol{U}_a|) \tag{3}$$

Following Bye (1985); Rooth and Xie (1992); Duhaut and Straub (2006); Gaube et al. (2015), if we assume that $|\boldsymbol{U_o}| << |\boldsymbol{U_a}|$, the stress difference can be approximated by neglecting quadratic terms in $|\boldsymbol{U_o}|$ as:

$$\boldsymbol{\tau}_{diff} = \boldsymbol{\tau} - \boldsymbol{\tau}_a \approx -2\rho_a C_D \,|\boldsymbol{U}_a|\,|\boldsymbol{U}_o|\,\boldsymbol{e} \tag{4}$$

where $\boldsymbol{e} = \cos[\theta]\boldsymbol{i} + 0.5\,\sin[\theta]\boldsymbol{j}$, with $(i, j)$ the local orthonormal basis having $\boldsymbol{i}$ oriented as $\boldsymbol{U_a}$ and $\theta$ the angle between the current and wind directions (See Fig. 1a). We next define $\alpha$, the angle between $\boldsymbol{U_o}$ and $\boldsymbol{e}$, defined by $\tan[\theta - \alpha] = \frac{1}{2}tan[\theta]$. Figure S1b shows that the absolute value of $\alpha$ can never exceed 19.5°, so roughly the directions of $\boldsymbol{U_o}$ and $e$ are close (*i.e.,* $\alpha \approx 0$) and $|\boldsymbol{U_o}|\boldsymbol{e} \approx |\boldsymbol{e}|\boldsymbol{U_o}$ with $|\boldsymbol{e}| = \frac{1}{2}\sqrt{1 + 3\cos[\theta]^2}$.

At mesoscale, we can consider that $\theta$, the angle between $\boldsymbol{U_a}$ and $\boldsymbol{U'_o}$ (where prime denotes the eddy part), has a random and uniform distribution between 0 and $2\pi$. $|\boldsymbol{e}|$ can therefore be approximated by its averaged value for $\theta$ ranging from 0 to $2\pi$: $\approx \frac{3}{4}$, *i.e.,* we can write:

$$\boldsymbol{\tau'}_{diff} \;\sim\; -\frac{3}{2}\rho_a C_D \,|\boldsymbol{U}_a|\,\boldsymbol{U'}_o\,. \tag{5}$$

## d. *Coupling Coefficient* $s_\tau$

$s_\tau$ is defined as the slope of the linear regression between surface stress curl and oceanic current vorticity and is evaluated at each grid point (0.25°) for the overlap period 2000-2008 using the surface geostrophic current from AVISO and the surface stress from QuikSCAT. The fields are first temporally averaged using a $29 - days$ running mean to suppress the weather-related variability Chelton et al. (2007), and the large-scale signal is removed using the high-pass Gaussian spatial filter with a 250 km cutoff (roughly corresponding to the eddy scale, as in *e.g.,* Seo (2017)) . Points with less than 80% of valid data in QuikSCAT or AVISO (mainly because of the presence of sea-ice) are flagged as missing values. Note $s_\tau$ is estimated over a range of conditions that implicitly includes the actual distribution of $\theta$ values, hence of $e$ values.

## e. *Uncertainties in the estimation of* $s_\tau$

The estimation of the coupling coefficient $s_\tau$ suffers from uncertainties in the observations, as *e.g.,* the smoothness of the AVISO product. To determine the $s_\tau$ sensitivity to the smoothness of AVISO, low-pass Gaussian spatial filter with a 67 km cutoff and 125 km cutoff are applied to the AVISO data. Then, $s_\tau$ is estimated as described in the previous section but using those low-pass filtered AVISO data ($s_{\tau 67}$, $s_{\tau 125}$). Figure 2 depicts the linear regression between the original $s_\tau$ and the low-pass filtered $s_{\tau 67}$, and $s_{\tau 125}$. Note the spatial pattern are very similar between $s_\tau$, $s_{\tau 67}$, and $s_{\tau 125}$: the correlation between $s_\tau$ and $s_{\tau 67}$ is 0.95 ($\sigma > 0.95$), and between $s_\tau$ and $s_{\tau 125}$ is 0.80 ($\sigma > 0.95$). The use of a smoother AVISO leads to a larger $s_\tau$, demonstrating the smoothness of AVISO can lead to an overestimation of $s_\tau$.

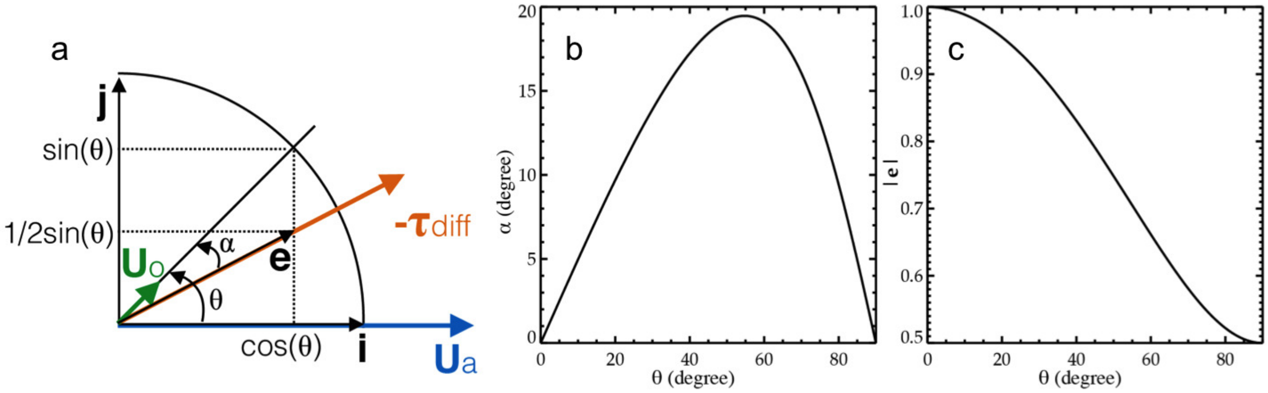


Fig. 1. (a): schematic representation of the surface current ($U_o$), the 10m-wind ($U_a$) and $-\tau_{diff}$ (*i.e.,* the opposite of the wind stress response to the oceanic currents). $\theta$ is the angle between the current and wind directions and $\alpha$ the angle between $U_o$ and $-\tau_{diff}$. (b) value of $\alpha$ according to $\theta$, both angles are in degree. (c) value of $|\boldsymbol{e}|$ according to $\theta$ (in degree). The Figure has been realized using IDL 8.6 (www.exelisvis.com/ProductsServices/IDL.aspx).

# References


Bye, J. A., 1985: Large-scale momentum exchange in the coupled atmosphere-ocean. *Elsevier oceanography series*, **40**, 51–61.

Chelton, D. B., Schlax, M. G., and Samelson, R. M., 2007: Summertime coupling between sea surface temperature and wind stress in the California Current System. *Journal of Physical Oceanography*, **37(3)**, 495–517.

Duhaut, T. H., and Straub, D. N., 2006: Wind stress dependence on ocean surface velocity: Implications for mechanical energy input to ocean circulation. *Journal of Physical Oceanography*, **36(2)**, 202–211.

Gaube, P., Chelton, D. B., Samelson, R. M., Schlax, M. G., and ONeill, L. W., 2015: Satellite observations of mesoscale eddy-induced Ekman pumping. *Journal of Physical Oceanography*, **45(1)**, 104–132.

Rooth, C., and Xie, L., 1992: Air-sea boundary layer dynamics in the presence of mesoscale surface currents. *Journal of Geophysical Research: Oceans*, **97(C9)**, 14431–14438.

Seo, H., 2017: Distinct Influence of Air–Sea Interactions Mediated by Mesoscale Sea Surface Temperature and Surface Current in the Arabian Sea. *Journal of Climate*, **30(20)**, 8061–8080.

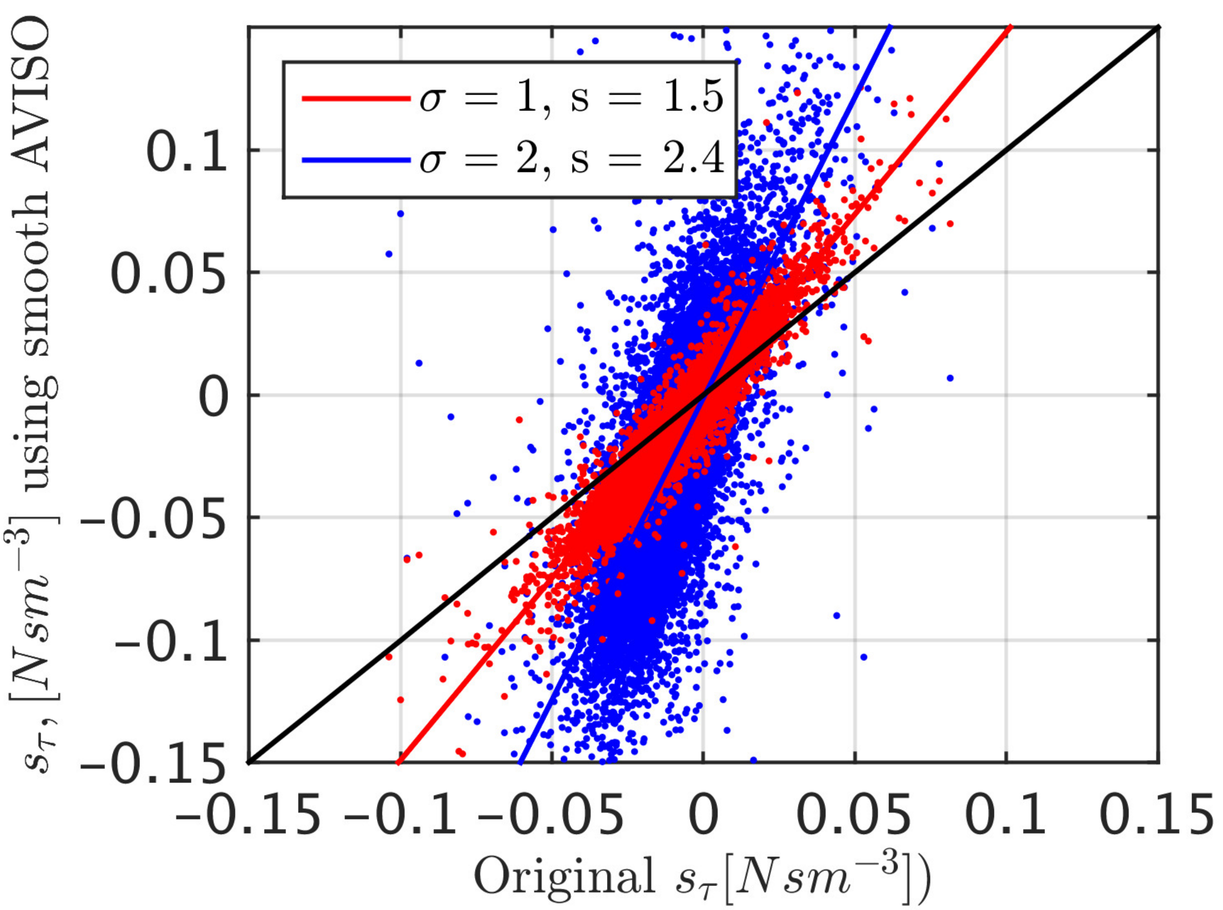


Fig. 2. Scatterplots between $s_\tau$ as estimated from AVISO and QuikSCAT and $s_\tau$ as estimated by first smoothing AVISO using a Gaussian spatial filter with a cutoff of about 67km (red) and 125km (blue). The black line highlights diagonal (with slope 1) whereas the red and blue lines indicate the linear regression of the corresponding scatterplots. The Figure has been realized using Matlab R2014b (https://www.mathworks.com/) and data from QuikSCAT V3 product (CERSAT, IFREMER) and E.U. Copernicus Marine Service Information data (AVISO).
